# Next-Generation Sequencing for Screening Analysis of Cystic Fibrosis: Spectrum and Novel Variants in a South–Central Italian Cohort

**DOI:** 10.3390/genes14081608

**Published:** 2023-08-11

**Authors:** Elisa De Paolis, Bruno Tilocca, Carla Lombardi, Maria De Bonis, Paola Concolino, Maria Elisabetta Onori, Claudio Ricciardi Tenore, Alessia Perrucci, Paola Roncada, Ettore Capoluongo, Andrea Urbani, Angelo Minucci, Concetta Santonocito

**Affiliations:** 1 Departmental Unit of Molecular and Genomic Diagnostics, Fondazione Policlinico Universitario A. Gemelli IRCCS, 00168 Rome, Italy; elisa.depaolis@policlinicogemelli.it (E.D.P.); maria.debonis@policlinicogemelli.it (M.D.B.); mariaelisabetta.onori@guest.policlinicogemelli.it (M.E.O.); claudio.ricciarditenore@guest.policlinicogemelli.it (C.R.T.); alessia.perrucci01@icatt.it (A.P.); andrea.urbani@policlinicogemelli.it (A.U.); angelo.minucci@policlinicogemelli.it (A.M.); 2 Department of Basic Biotechnological Sciences, Intensivological and Perioperative Clinics, Catholic University of Sacred Heart, 00168 Rome, Italy; carla.lombardi01@icatt.it; 3 Department of Health Science, University “Magna Graecia” of Catanzaro, 88100 Catanzaro, Italy; tilocca@unicz.it (B.T.); roncada@unicz.it (P.R.); 4 Department of Molecular Medicine and Medical Biotechnologies, University Federico II, 80138 Naples, Italy; ettore.capoluongo@aoec.it; 5 Department of Clinical Pathology and Genomics, Ospedale Cannizzaro, 95021 Catania, Italy

**Keywords:** cystic fibrosis, cystic fibrosis transmembrane conductance regulator gene, CFTR, next-generation sequencing, cystic fibrosis carriers

## Abstract

The incidence of cystic fibrosis (CF) and the spectrum of cystic fibrosis transmembrane conductance regulator (CFTR) gene variants differ among geographic regions. Differences in CF carrier distribution are also reported among Italian regions. We described the spectrum of the CFTR variants observed in a large group of subjects belonging from central–southern Italy. We also provide a predictive evaluation of the novel variants identified. CFTR screening was performed in a south–central Italian cohort of 770 subjects. We adopted a next-generation sequencing (NGS) approach using the Devyser CFTR NGS kit on the Illumina MiSeq System coupled with Amplicon Suite data analysis. Bioinformatics evaluation of the impact of novel variants was described. Overall, the presence of at least one alternative allele in the CFTR gene was recorded for 23% of the subjects, with a carrier frequency of CF pathogenic variants of 1:12. The largest sub-group corresponded to the heterozygous carriers of a variant with a conflicting interpretation of pathogenicity. The common CFTR p.(Phe508del) pathogenic variants were identified in 37% of mutated subjects. Bioinformatics prediction supported a potential damaging effect for the three novel *CFTR* variants identified: p.(Leu1187Phe), p.(Pro22Thr), and *c.744-3C > G*. NGS applied to CF screening had the benefit of: effectively identifying asymptomatic carriers. It lies in a wide overview of CFTR variants and gives a comprehensive picture of the carrier prevalence. The identification of a high number of unclassified variants may represent a challenge whilst at the same time being of interest and relevance for clinicians.

## 1. Introduction

Cystic fibrosis (CF, OMIM 219700) is a multisystem involvement genetic disease mainly affecting the intestinal and respiratory systems. The molecular basis of CF lies in the occurrence of mutations in the cystic fibrosis transmembrane conductance regulator (*CFTR*) gene, with an autosomal recessive inheritance [1]. The *CFTR* gene is located on the long arm of chromosome 7 (7q31.2) and consists in 27 exons encoding for an epithelial cell protein of 1480 amino acids belonging to the ATP Binding Cassette family [2]. The major biological role of the CFTR protein consist in the transmembrane transport regulation of chlorine and other anions using the cellular ATP [3]. Several epithelial cell types express the CFTR protein, mainly in the airways, digestive system, sweat glands, and genitourinary tract. It is also found at lower levels in non-epithelial cells and in tissues not directly involved in the CF disease, such as the cornea and vascular endothelium [4].

The incidence of CF and the distribution and frequency of *CFTR* gene mutations differ among geographic regions and ethnic groups. Overall, the incidence of CF in the Caucasian population is approximately 1:2500–3500 neonates/year [5]. In Italy, data show an incidence of CF ranging between 1:4854 and 1:2438 [6,7]. In addition, data regarding the CF carrier frequency differs among Italian regions, with the north-eastern population characterized by the highest estimated incidence reported so far [8].

*CFTR* gene testing can be performed for diagnostic or screening purposes. Diagnosis of CF is based on the combination of clinical manifestations with the finding of abnormal CFTR, according to validated diagnostic assays such as the immunoreactive trypsinogen test (IRT), the sweat test, and genetic analysis. On the other hand, carrier screening evaluation is performed: (1) in subjects that are close relatives of a CF patient; (2) in partners of individuals carrying a CF mutation; (3) prenatally if parents are CF carriers; (4) in the context of national screening programs. An increasing trend in *CFTR* molecular testing in couples without a CF family history has been observed worldwide [9,10,11]. Since 1997, guidelines from the National Institutes of Health have recommended CF carrier testing to all couples planning a pregnancy [12]. Population screening by genetic testing has the benefit of identifying heterozygous adults and allowing informed reproductive choices [13]. Several screening approaches have been adopted, with differences in testing methodologies. In contrast to older genetic tests, which included pre-set panels of the most common *CFTR* mutations with reference to a specific population, the introduction of high-throughput technologies such as next-generation sequencing (NGS) has allowed the effective analysis of the entire *CFTR* gene. Consequently, NGS plays a relevant role in the implementation of preventive strategies and corrective therapies, overcoming population bias [14]. To date, more than 2000 different variants in the *CFTR* gene have been identified according to Clinvar database [15] and Cystic Fibrosis Mutation Database [16]. Among these, up to 80% of the CF cases are related to the presence of the deleterious mutation ∆F508 (c.1521_1523delCTT, p.Phe508del) [16].

This study has as its primary aim the evaluation of the frequency and type of *CFTR* variants observed in a large group of healthy subjects from central and southern Italy who underwent molecular screening testing of the *CFTR* gene at our institution as the referral center. The molecular investigation was performed by using a full-coding NGS approach, allowing us to obtain a broad overview of the variant distribution and a picture of *CFTR* carriers in this geographical region. We additionally speculate about the pathogenicity of *CFTR* novel variants detected in our cohort, in order support their classification. To the best of our knowledge, this study involved the largest cohort of subjects coming from south–central Italy and screened for *CFTR* alteration using an NGS approach.

## 2. Materials and Methods

### 2.1. Patients

This was a retrospective single-center study performed at the Policlinico “A. Gemelli” Foundation in Rome. From January 2015 to December 2021, a total of 770 unaffected and unrelated subjects from central–southern Italy were screened for genetic analysis of the *CFTR* gene in the context of prenatal, male infertility or medically assisted pregnancy counselling. CFTR carriers enrolled in the study had the following characteristics: 58.5% female, 41.5% males, and Caucasian of central or southern Italian origin (self-declared).

The present study was in accordance with the Declaration of Helsinki, and the evaluated patients were included in the protocol ID 4208 approved by the Ethics Committee of Gemelli Hospital Foundation. Informed consent was obtained from each participant.

Starting from the entire cohort of 770 subjects, we described, for the purpose of this study, the carriers of *CFTR* variants classified as pathogenic/likely pathogenic, with conflicting interpretation of pathogenicity (CIP), variants of unknown significance (VUS), and previously unreported (novel).

### 2.2. DNA Extraction and Next-Generation Sequencing

DNA was extracted from whole blood samples using the QIAmp DNA Mini kit on the Qiacube instrument (Qiagen, Milan, Italy). The quantitation of the extracted DNA was performed using the Qubit dsDNA BR fluorometric assays (Life Technologies, Gaithersburg, MD, USA). The purity and quality of the extracted DNA were assessed by using a spectrophotometer method. CFTR full gene screening was performed using the amplicon-based Devyser CFTR NGS kit (Devyser, Stockholm, Sweden). Briefly, for each analyzed sample, sequencing libraries were prepared starting from 10 ng of DNA, obtained according to the manufacturer’s protocol. DNA libraries were generated using a two-step protocol that allows target multiplex amplification and the incorporation of the Illumina adapters. After a magnet bead purification step, the quantitation of the final pool of libraries was performed using Qubit dsDNA HS fluorometric assays (Life Technologies, Gaithersburg, MD, USA). Sequencing reaction was carried out on the Illumina MiSeq System (Illumina, San Diego, CA, USA) in paired-end read mode (2 × 151 cycles).

### 2.3. NGS Data Analysis and Interpretation

Data analysis was performed in order to detect *CFTR* single-nucleotide variants (SNVs), insertions/deletions (indels), and copy number variation (CNV). The FastQ data obtained were analyzed using CE-IVD Amplicon Suite Software (SmartSeq, Novara, Italy). Variants with a mean depth of coverage below 100x were excluded from the evaluation. Pre-classification of genomic variants was obtained according to the American College of Medical Genetics and Genomics guidelines, and all the sequence variants identified were named according to Human Genome Variation Sequence nomenclature. ClinVar [14], CFTR-France [17], CFTR2 [18], LOVD [19], VarSome [20], and Intervar [21] were used for the final classification of the variants.

Previously unreported variants were defined as “novel”, and the impact of each missense sequence mutation was predicted using the CYSMA biological tool [22]. This tool computes the impact of the sequence variation in terms of Ortholog conservation, shared domain conservation, secondary structure analysis and 3D analysis forecasting [23]. Analogous observations have also been computed to assess the impact of the sequence variation on the protein structure. In this light, the high-definition 3D structure of the wild-type protein (UniProt accession number: P13569) was retrieved from the Protein Data Bank Database [24] under the accession number 5AUK. This, in turn, was used as the input structure for the modelling of each variants’ structure through Swiss Model [25]. Both wild-type and mutant structures were finally used as the input information to feed the Dynamut2 bioinformatic tool [26]. This tool comparatively evaluates pairs of proteins (i.e., the wild-type protein versus the mutated counterpart) in order to predict the stabilizing/destabilizing effect of the mutation, by considering the physical and chemical interactions occurring among the amino acid residues of the protein, the distance between residues, and the protein folding [27]. The biological impact of the amino acid substitution following the sequence mutation was computed via PolyPhen2 and SIFT [28,29,30]. Prediction of the slicing effect was assessed using Human Splicing Finder [31] and MobiDetails [32].

## 3. Results

### 3.1. Overall Description of CFTR Mutational Spectrum

A total of 770 unaffected and unrelated subjects screened in our institution for *CFTR* mutations participated in this study. The presence of at least one alternative allele in the *CFTR* gene was recorded for 23% of the subjects (177/770 screened subjects). Particularly, 159 individuals were diagnosed as heterozygous carriers of one pathogenic/likely pathogenic variant (n = 57; 37%), CIP variant (n = 76; 49.3%), VUS (n = 18; 11.7%) or previously unreported variant (n = 3; 2%). A total of 18 individuals were diagnosed as carriers of the following CFTR complex alleles: p.(Gly576Ala)/p.(Arg668Cys) (n = 8); p.(Gly576Ala)/p.(Arg668Cys)/p.(Arg75Gln) (n = 1); p.(Phe508del)/p.(Arg668Cys) (n = 1); p.(Phe508del)/p.(Asn1303Lys) (n = 1); p.(Ala455Val)/c.2620-15C>G (n = 1); p.(Ala455Val)/p.(Leu997Phe) (n = 1); p.(Arg31Cys)/p.(Ala455Val) (n = 1); p.(Arg75Gln)/p.(Ala455Val) (n = 1); c.2490+44A>C/p.(Ala455Val) (n = 1); p.(Leu967Ser)/p.(Glu1418Argfs*14) (n = 1), and p.(Leu1077Pro)/p.(Asp192Gly) (n = 1). All these *CFTR* complex alleles were considered of unknown significance given the lack of the cis/trans status data, with the exception of the p.(Gly576Ala)/p.(Arg668Cys) reported as likely benign (ClinVar ID 916697, accessed June 2023) (Supplementary Table S1).

**Figure 1:**
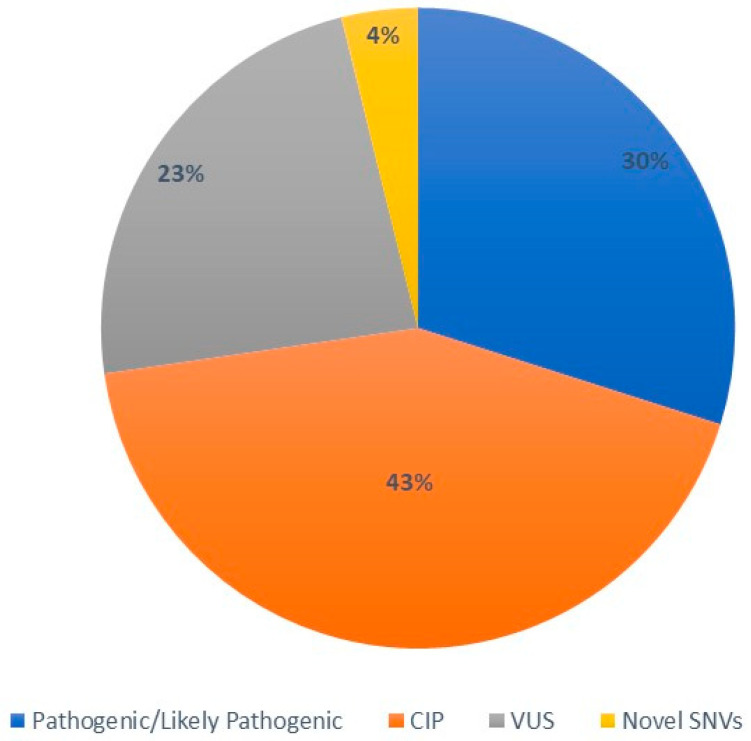
Classification of *CFTR* variants. The pie chart shows the different groups of *CFTR* alterations identified in the cohort of the study, sliced by color. Annotations were in accordance with ClinVar database (accessed on 15 June 2023). CIP: conflicting interpretation of pathogenicity; VUS: variants of uncertain significance; SNVs: single-nucleotide variants.

Overall, 77 unique *CFTR* variants were found, classifiable as: 23 pathogenic/likely pathogenic variants, 33 CIP, 18 VUS, and 3 novel variants (according to ClinVar database, last accessed April 2023) (Figure 1).

**Figure 2:**
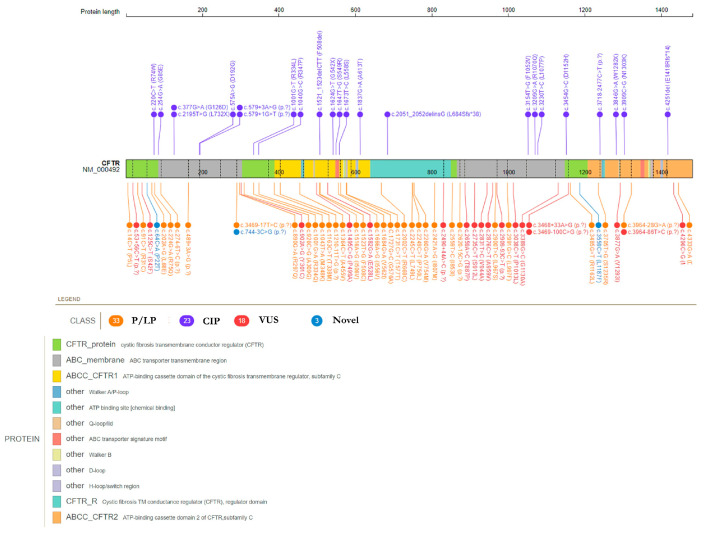
Distribution of *CFTR* identified variants in the context of protein structure. The figure shows the linear map of the *CFTR* gene (NM_000492) and the exon/intron location of the genetic variants. Pathogenic and likely pathogenic variants are reported above (purple). Variants of uncertain significance (VUS, red), variants with conflicting interpretation of pathogenicity (CIP, orange), and novel single-nucleotide variants (blue, SNVs) are reported below. Protein domains are represented by different colored areas (https://proteinpaint.stjude.org/ (accessed on 15 June 2023)).

The identified *CFTR* variants were distributed along the entire sequence of the *CFTR* gene, affecting all the main protein domains (Figure 2).

### 3.2. CFTR Pathogenic/Likely Pathogenic Variants

Of the screened subjects tested in the present study, 61 resulted at risk of the transmission of a pathogenic/likely pathogenic *CFTR* allele (61/770, 8%), with an overall carrier frequency of 1:12.

All the 23 detected *CFTR* variants annotated as pathogenic/likely pathogenic in the ClinVar repository (last accessed on 15 June 2023) are collected in Table 1. Among these, the highest prevalence of the c.1521_1523delCTT, p.(Phe508del) pathogenic variants (rs113993960) emerged, as well-known CF characteristic alterations. This common CFTR mutation was detected in a total of 23 screened subjects, with a frequency of 37% (23/61) among all the pathogenic/likely pathogenic variant carriers. Also, from the evaluation of the entire cohort of subjects, carriers of an alternative CFTR allele, p.(Phe508del), resulted the most frequent (13% (23/177)).

**Figure 3:**
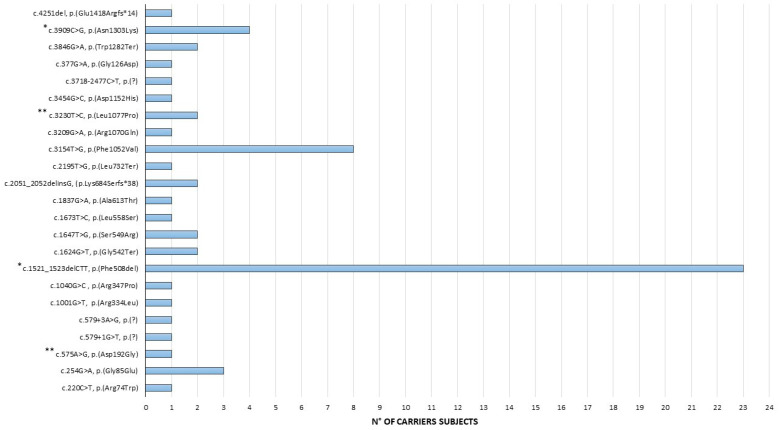
Pathogenic and likely pathogenic CFTR sequence variants (n = 23) distribution among the 62 carriers identified in our cohort. * One carrier of the complex allele: p.(Phe508del)/p.(Asn1303Lys); ** one carrier of the complex allele: p.(Leu1077Pro)/p.(Asp192Gly).

We also identified the following as recurrent pathogenic alterations: c.3154T>G, p.(Phe1052Val) (8/177, 4.5%); c.3909C>G, p.(Asn1303Lys) (4/177, 2%); and c.254G>A, p.(Gly85Glu) (3/177, 1.7%). All the other detected variants resulted in a frequency below 1% in our cohort (Figure 3).

### 3.3. CFTR Variants with Conflicting Interpretation of Pathogenicity and Variants of Uncertain Significance

Among the screened subjects, the largest mutational sub-group corresponded to the heterozygous carriers of a variant classifiable as CIP, with a total of 33 different *CFTR* variants identified (Table 2). In this sub-group, the highest prevalence resulted in c.2991G>C, p.(Leu997Phe) (14/177, 8%), c.2620-15C>G, p.? (12/177, 7%), and c.2002C>T, p.(Arg668Cys) (12/177, 7%).

**Figure 4:**
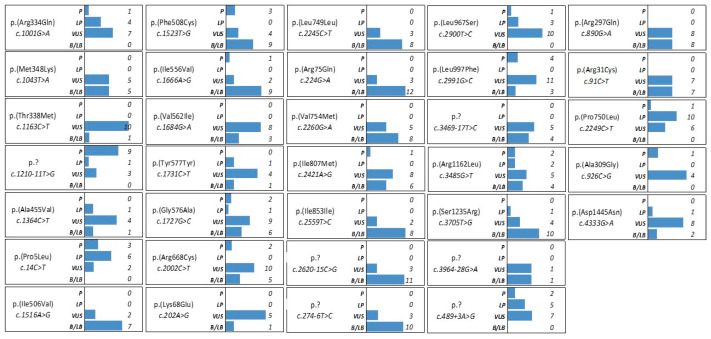
Details of the ClinVar interpretations for each variant with conflicting interpretation of pathogenicity identified in the study (accessed on 15 June 2023).

Figure 4 describes the distribution of the different ClinVar interpretations collected for each CIP variant identified (accessed, 15 June 2023). Some of the *CFTR* variants reported to have a conflicting interpretation of pathogenicity have an overall number of annotations strongly biased toward a pathogenic/likely pathogenic significance such as: c.1210-11T>G, p.(?) (10 annotations as pathogenic/likely pathogenic variant vs. 3 annotations as VUS), the c.14C>T, p.(Pro5Leu) (9 annotations as pathogenic/likely pathogenic variant vs. 2 annotations as VUS), and the c.2249C>T, p.(Pro750Leu) (11 annotations as pathogenic/likely pathogenic variant vs. 6 annotations as VUS).

Additionally, 18 CFTR VUS were identified in 23 subjects (23 out of 177, 13%), with the CFTR VUS c.125C>T, p.(Ser42Phe) (rs143456784) and the c.2909-93C>T, p.(?) (rs144455881) identified in a total of 3 unrelated individuals/each. Table 3 presents a list of VUS with details about their annotations in the main reference databases including CFTR-France and CFTR2. For each variant, we also reported information about its functional effects, as predicted from several bioinformatic tools. In addition, we reported as VUS five *CFTR* alterations with an associated record in dbSNP (https://www.ncbi.nlm.nih.gov/snp/ (accessed on 15 June 2023)) and gnomAD (https://gnomad.broadinstitute.org/ (accessed on 15 June 2023)) databases and without a clinical annotation in the abovementioned databases (ClinVar, CFTR-France, CFTR2, and LOVD). Among these, four *CFTR* alterations affect non-canonical splice sites: c.2490+44A>C, c.2909-93C>T, c.3469-100C>G, and c.3964-86T>C. The nucleotide changes are located in deep intronic regions and were predicted to not significantly affect *CFTR* splicing processes. Indeed, from the bioinformatics prediction of pathogenicity for the *CFTR* c.53-56C>T variant, an intermediate effect on splicing emerged, with a predicted activation of a cryptic donor site with potential alteration of splicing.

### 3.4. CFTR Novel Variants

In this study, a total of three previously unreported *CFTR* alterations were identified in three individuals. In particular, we detected: two novel missense variants (c.3559C>T, p.(Leu1187Phe); c.64C>A, p.(Pro22Thr)) and one novel splicing variant (c.744-3C>G, p.(?)).

**Figure 5:**
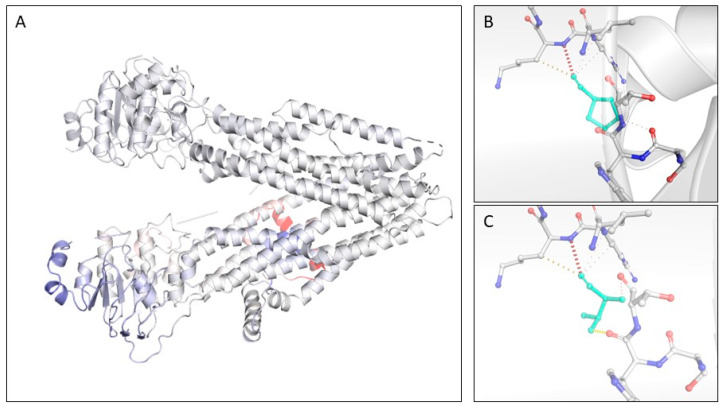
(**A**): Tridimensional structure of the CFTR protein as of the 5AUK structure in PDB data repository. Protein moieties are colored according to the vibrational entropy change upon mutation 22 Pro → Thr. Blue shades are representative of a rigidification of the structure while red shades indicate a gain in flexibility. Interatomic interactions of the wild-type Pro22 (**B**) and mutant Thr22 (**C**) protein. Wild-type and mutant residues are colored in light green and are represented along with the surrounding residues that are involved in any type of interaction.

*In silico* evaluation of the protein mutation revealed a particular scenario for each of the novel missense mutations considered in the study. Alteration of the protein CFTR through the substitution of the amino acid Proline with a Threonine in position 22 (p.Pro22Thr) has not been previously reported in the gnomAD or in ClinVar. The wild-type residue Pro22 is conserved at 98% among the CFTR orthologs, and the Pro22Thr mutation has never been observed in other species. Caenorhabditis elegans manifests the Phe-residue instead of the Pro. Regarding the CFTR structure, the mutation is predicted, with a score of 0.96, to fall in an α-helix structure of the N-terminal region of the protein, a cytosolic region, also called the “lasso motif” because of its shape. Here, the first 40 amino acid residues are partially inserted into the membrane, while the end portion forms the “lasso” helix. Conservation of the wild-type amino acid among the homolog domain is computed at 61.79%, whereas the mutant domain is found in 3.25% of the N-terminal homologs. Prediction of the effects of the p.(Pro22Thr) mutation has been accomplished using the high-definition 3D structure available in the Protein DataBank under accession 5AUK. Prediction of the thermodynamic stability of the protein upon mutation revealed a weak destabilizing effect for P22T mutation with a ΔΔG: −0.065 kcal/mol. The 3D structures predict that the replacement of a proline is likely to increase the flexibility of the region as reported by the Δ Vibrational Entropy Energy Between Wild-Type and Mutant of +0.031 kcal·mol^−1^·K^−1^. A visual representation of the ΔVibrational Entropy Energy is reported below (Figure 5, panel A). Concerning the solvent accessibility, both the wild-type Pro22 and the mutant p.(Pro22Thr) are predicted to be exposed to the outer layer. The two residues have a different polarity, which could interfere with hydrogen-bonding capabilities. The mutant residue is predicted to form more hydrogen bonds and less hydrophobic interactions than the wild type. The mutant residue is not predicted to introduce steric clashes (Figure 5, panels B,C). Additionally, prediction of the possible impact of an amino acid substitution on the structure and function of a human protein accomplished by PolyPhen-2 categorizes this mutation as damaging with a score of 1; on the other hand, SIFT prediction based on sequence homology and the physical properties of amino acids label the mutation as tolerated based on a score of 0.15.

Mutation of the protein CFTR through a substitution of the amino acid Leucine with a Phenylalanina in the position 1187 (p.Leu1187Phe) has not been previously reported in the gnomAD or in ClinVar. The wild-type residue Leu1187 is conserved at 76% among the CFTR orthologs and the p.(Leu1187Phe) mutation is detected in 4% orthologues. Bos taurus and Ovis aries show the Pro-residue instead of the Leu1187. Gallus gallus and Taeniopygia guttata are featured by the Phe-residue, Tetraodon nigroviridis and Takifugu rubripes displaying the Gly, and Mus musculus and Rattus norvegicus are characterized by Ser residues. On the other hand, Ornithorhynchus anatinus, Danio rerio and Oryzias latipes have shown Ile, Lys, and Gln residues instead of the wild-type Leu1187. Regarding the CFTR structure, the mutation is predicted, with a score of 0.845, to fall in a loop region of the membrane-spanning domain 2 (MSD2) domain of the CFTR protein. Conservation of the wild-type amino acid among the homolog domain is computed at 29.92%, whereas the mutant domain is found in 1.57% of the MSD2 homologs. Prediction of the effects of the p.(Leu1187Phe) mutation cannot be accomplished with the high-definition 3D structure available in the Protein DataBank under accession 5AUK since the available structure fails to model the sequence region involved by the present mutation. Prediction of the protein structure allows the creation of a 3D model suitable for the prediction of the thermodynamic stability of this missense mutation. Such prediction is run on the DUET tool (http://biosig.unimelb.edu.au/duet/stability_prediction (accessed on 15 June 2023)), supporting its own PBD structure as the input. The p.(Leu1187Phe) mutation is predicted to be destabilizing with a ΔΔG of −1.171kcal/mol. Prediction of the possible impact of an amino acid substitution on the structure and function of a human protein accomplished by PolyPhen-2 categorizes this mutation as benign with a score of 0.001; on the other hand, SIFT prediction based on sequence homology and the physical properties of amino acids label the mutation as tolerated based on a score of 0.72.

Among the novel *CFTR* alterations, one variant affects non-canonical splice sites. The prediction analysis of the CFTR c.744-3C>G variant supported its deleterious effect, with the breaking of a wild-type acceptor site and the activation of a new acceptor site within intron 6.

## 4. Discussion

The aims of the present study were to (1) describe the CF carrier population of central and southern Italy and referred to our institution and (2) characterize the *CFTR* alterations identified, defining the type and frequency.

CF is the most common autosomal recessive disease in the Caucasian population. The *CFTR* allele variability is high, with variants distributed throughout the entire gene. The heterogeneity also emerged in terms of gene variant clinical consequences that are still uncertain for many *CFTR* variants [33]. Nucleotide sequence changes are mainly located in the coding regions, with the prevalence of missense type (40%), followed by frameshift (16%), nonsense (8%), large indels (3%), and in-frame indels (2%). Splicing variants represent approximately 12% of all *CFTR* alterations. The classification of the *CFTR* mutations depends on the functional effect on CFTR protein, with six different classes. Particularly, class I, II, and III mutations are associated with a more severe phenotype, with higher incidence of meconium ileus, pancreatic insufficiency, malnutrition, early and severe deterioration of lung function, and severe liver disease. Classes IV and V are associated with mild lung disease, preserved pancreatic function and longer life expectancy, and tend to be phenotypically dominant if they occur in association with class I–III mutations [34].

Among the Italian regions, a CF prevalence variability was observed, from a minimum of 4.3 per 100,000 inhabitants in the Friuli-Venezia Giulia region (northern Italy), to a maximum of 10.2 per 100,000 inhabitants in the Basilicata region (southern Italy). Considering the 10 central and southern Italian regions (including Sicily), the prevalence spans from the highest one, Basilicata, to 4.9 per 100,000 inhabitants in the Campania region (mean prevalence of 7.4 per 100,000 inhabitants) [35]. Similarly, the frequency of healthy CF carriers of a single mutation is estimated to be 1:25 in the Caucasian general population and is concordant with Italian carrier screening data. Differences among Italian regions are reported, with a frequency of 1:31 in northern Italy [8], 1:27 in the Lazio region (central Italy) [5], 1:16 in Sicily [34], and 1:14 in the Basilicata region [36] (southern Italy). In the present paper, we calculated a frequency of CF carriers of 1:12 (8%), which is higher than that expected for the Caucasian population and consistent with the studies of Chamayou et al. (6%), analyzing CF carriers in Sicily using an NGS approach [33], and Dell’Edera et al., analyzing Basilicata CF carriers using whole-gene analysis (7%) [36]. We identified the typical *CFTR* p.(Phe508del) mutation in 37% of pathogenic variant carriers. This result was higher than the one reported for Sicilian CF carriers (30%) and lower than the Italian data (45%) [34]. Among the other pathogenic *CFTR* variants identified in our cohort, we confirmed the high frequency of p.(Asn1303Lys) and p.(Gly85Glu) in the Italian CF population. *CFTR* mutations frequent in the northern Italian regions, such as c.621+1G>T, p.(Ile507del), p.(Gly551Asp), and p.(Arg1162Ter), were absent in our population [37]. Of note, epidemiological data and *CFTR* mutation distribution reported in literature are not fully comparable among the different studies due to several biases. In our opinion, one of the most relevant differences that emerged from the *CFTR* molecular studies is the type of genetic test performed on affected or carrier subjects, which include screening for a small panel of the most common mutations and also whole-gene sequencing. In order to obtain a high detection rate in the CF screening program, population-specific mutation panels can be considered. In these cases, panels should include at least the prevalence of approximately 85% of the *CFTR* mutations detected in the specific population, according to the Italian Society for the Study of Cystic Fibrosis [38]. Additionally, the availability of sequencing tests characterized by a greater sensitivity (mutation detection rate of 99%) such as the NGS applied to whole-gene analysis, makes use of extended approaches, which are more effective. In this context, in the ever-expanding number of countries with heterogeneous populations, the use of mutation panels could lead to CF underestimation or misdiagnosis. On the other hand, considering that a small portion of all the known CFTR variants are, to date, ranked, the widespread adoption of NGS is undoubtedly leading to the identification of additional new variants, expanding the overall number of *CFTR* alterations with uncertain significance. In the case of novel or rare variants, often classified as CIP or VUS, their inclusion in a described *CFTR* mutational class is a challenge. In this paper, we reported the CIP subgroups of variants as the most represented. Evaluating the significance for each CIP variant as reported in the ClinVar database, we underlined that some of these unclassifiable variants may deserve attention, having depositions that support a certain degree of pathogenicity, such as c.1210-11T>G, p.(Pro5Leu), and c.489+3A>G (Figure 4). In the cohort of screened subjects, we identified three novel CFTR variants, including one intronic nucleotide change. The in silico evaluations adopted here relied on the querying of multiple and independent algorithms. The registered independent observations support each other in the definition and characterization of the novel variants identified. Among these, the in silico analyses supported the deleterious effect of the novel *CFTR* c.744-3C>G splicing variants identified as rare CFTR alterations in the cohort (one subject). Moreover, concordance in the results was observed when evaluating the missense novel mutations on the basis of the sequence variation and the effects on the protein structure, supporting the accuracy and likelihood of the computations that are, all the same, deserving of experimental confirmation (evaluating, for example, mRNA and protein levels compared to other *CFTR* pathogenic variants such as the common p.(Phe508del)).

We underline that studies investigating the many aspects related to CF carriers are currently ongoing. These investigations will clarify several molecular and clinical topics, allowing a deeper understanding of the potential harms and benefits.

The practical value of the CF screening program adopted to identify *CFTR* heterozygous carriers primarily consists in supporting responsible procreative choices and paying attention to the CF occurrence in newborns. In these contexts, the identification of unclassifiable *CFTR* variants should also raise relevant clinical issues. This is particularly the case when unclear *CFTR* genotyping occurs in the context of newborn screening. In such situations, both the families and the growing child may have to cope with unhelpful uncertainty, and the role of clinicians and geneticists becomes pivotal. All efforts should be directed towards the continuous counselling of these families.

In addition, an open debate concerns the pathophysiological consequences of having only one *CFTR* functional copy, with an estimated 50% of protein function. This protein expression level is generally considered sufficient to maintain a healthy condition. However, several studies have underlined that CF carriers can have a significantly increased risk for CF-related conditions in multiple organ systems such as chronic bronchitis and bronchiectasis, male infertility, and pancreatitis [39,40]. Even if most of the CF carriers are asymptomatic, it appears plausible that selected heterozygous carriers undergo a reduction in the normal CFTR protein function as a response to environmental factors or epigenetic regulation, developing clinical manifestations [41].

The present study reported a high number of detected unique *CFTR* variants (n = 77), with novel alterations (n = 3) identified and characterized. The overall frequency of carriers of pathogenic/likely pathogenic *CFTR* (8%, 1:12) was consistent with the previously reported data regarding the southern Italian region and NGS-based *CFTR* analysis. We additionally underlined that the identification, reporting, and monitoring of *CFTR* CIP and VUS carriers could be of interest for clinicians and medical geneticists.

Overall, clinicians and patients or asymptomatic subjects may benefit from *CFTR* NGS mutational analysis. Beyond the well-known clinical implications of CF diagnosis in a perinatal program or in a preconceptional assessment, clinicians could also better monitor the unrevealed CF-related conditions, with more effective preventive approaches for asymptomatic carriers. In addition, healthy subjects that are informed of being CF carriers could be motivated to avoid other at-risk factors (e.g., alcohol in pancreatitis prevention). A high-throughput sequencing approach supports an effective CFTR screening analysis and CF molecular diagnosis, given the possibility to avoid population and epidemiological biases, even if custom panels have proven to have a high detection rate. In the case of NGS adoption, researchers and clinicians should be willing to make additional efforts towards variant classification and ranking in order to support and encourage advances in CF diagnosis and therapeutic chances.

## Figures and Tables

**Table 1:** *CFTR* sequence variants classified as pathogenic/likely pathogenic identified in our cohort (transcript, NM_000492.4).

HGVS cDNA Change	Protein Change	dbSNP
c.220C>T	p.(Arg74Trp)	rs115545701
c.254G>A	p.(Gly85Glu)	rs75961395
c.377G>A	p.(Gly126Asp)	rs397508609
c.575A>G	p.(Asp192Gly)	rs397508758
c.579+1G>T	p.(?)	rs77188391
c.579+3A>G	p.(?)	rs397508761
c.1001G>T	p.(Arg334Leu)	rs397508137
c.1040G>C	p.(Arg347Pro)	rs77932196
c.1521_1523delCTT	p.(Phe508del)	rs113993960
c.1624G>T	p.(Gly542Ter)	rs113993959
c.1647T>G	p.(Ser549Arg)	rs121909005
c.1673T>C	p.(Leu558Ser)	rs193922504
c.1837G>A	p.(Ala613Thr)	rs201978662
c.2051_2052delinsG	p.(Lys684Serfs*38)	rs121908799
c.2195T>G	p.(Leu732Ter)	rs397508609
c.3154T>G	p.(Phe1052Val)	rs150212784
c.3209G>A	p.(Arg1070Gln)	rs78769542
c.3230T>C	p.(Leu1077Pro)	rs139304906
c.3454G>C	p.(Asp1152His)	rs75541969
c.3718-2477C>T	p.(?)	rs75039782
c.3846G>A	p.(Trp1282Ter)	rs77010898
c.3909C>G	p.(Asn1303Lys)	rs80034486
c.4251del	p.(Glu1418Argfs*14)	rs397508706

Footnotes: *CFTR*, cystic fibrosis transmembrane conductance regulator; dbSNP, single-nucleotide polymorphism database.

**Table 2:** *CFTR* sequence variants classified with a conflicting interpretation of pathogenicity in our cohort (transcript, NM_000492.4).

HGVS cDNA Change	Protein Change	dbSNP
c.14C>T	p.(Pro5Leu)	rs193922501
c.91C>T	p.(Arg31Cys)	rs1800073
c.202A>G	p.(Lys68Glu)	rs397508332
c.224G>A	p.(Arg75Gln)	rs1800076
c.274-6T>C	p.(?)	rs371315549
c.489+3A>G	p.(?)	rs377729736
c.890G>A	p.(Arg297Gln)	rs143486492
c.926C>G	p.(Ala309Gly)	rs397508818
c.1001G>A	p.(Arg334Gln)	rs397508137
c.1043T>A	p.(Met348Lys)	rs142920240
c.1163C>T	p.(Thr338Met)	rs143860237
c.1210-11T>G	p.(?)	rs73715573
c.1364C>T	p.(Ala455Val)	rs74551128
c.1516A>G	p.(Ile506Val)	rs1800091
c.1523T>G	p.(Phe508Cys)	rs74571530
c.1666A>G	p.(Ile556Val)	rs75789129
c.1684G>A	p.(Val562Ile)	rs1800097
c.1731C>T	p.(Tyr577=)	rs55928397
c.1727G>C	p.(Gly576Ala)	rs1800098
c.2002C>T	p.(Arg668Cys)	rs1800100
c.2245C>T	p.(Leu749Leu)	rs151235408
c.2249C>T	p.(Pro750Leu)	rs140455771
c.2260G>A	p.(Val754Met)	rs150157202
c.2421A>G	p.(Ile807Met)	rs1800103
c.2559T>C	p.(Ile853Ile)	rs1800104
c.2620-15C>G	p.(?)	rs139379077
c.2900T>C	p.(Leu967Ser)	rs1800110
c.2991G>C	p.(Leu997Phe)	rs1800111
c.3469-17T>C	p.(?)	rs79718042
c.3485G>T	p.(Arg1162Leu)	rs1800120
c.3705T>G	p.(Ser1235Arg)	rs34911792
c.3964-28G>A	p.(?)	rs397508651
c.4333G>A	p.(Asp1445Asn)	rs148783445

Footnotes: *CFTR*, cystic fibrosis transmembrane conductance regulator; dbSNP, single-nucleotide polymorphism database.

**Table 3:** *CFTR* sequence variants classified as VUS with details about annotations in the main databases and predicted functional effects on protein (NM_000492.4). The IVS name was reported for the intronic *CFTR* variants.

HGVS cDNA Change	Protein Change	N° of Carriers	db SNP	CFTR-France ^a^	CFTR2 ^b^	LOVD ^c^	InterVar ^d^	Varsome ^e^
c.125C>T	p.(Ser42Phe)	3	rs143456784	VUS	n/a	P/VUS	VUS	VUS
c.902A>G	p.(Tyr301Cys)	1	rs150691494	VUS	n/a	VUS	VUS	VUS
c.1495C>G	p.(Pro499Ala)	1	rs397508219	n/a	n/a	n/a	VUS	LP
c.1582G>A	p.(Glu528Lys)	1	rs773018372	n/a	n/a	n/a	VUS	VUS
c.2659A>C	p.(Thr887Pro)	1	rs770359007	n/a	n/a	n/a	LB	VUS
c.2735C>T	p.(Ser912Leu)	1	rs121909034	VUS	VUS	VUS	B	LB
c.2831T>C	p.(Val944Ala)	1	rs141747560	n/a	n/a	n/a	VUS	LP
c.2876C>T	p.(Ala959Val)	1	rs397508448	VUS	n/a	n/a	VUS	LP
c.3038C>T	p.(Pro1013Leu)	1	rs193922516	VUS	n/a	VUS	VUS	LP
c.3389G>C	p.(Gly1130Ala)	1	rs397508550	n/a	n/a	n/a	VUS	LP
c.3468+33A>G	p.(?)	1	rs1792459342	n/a	n/a	n/a	n/a	VUS
c.3877G>A	p.(Val1293Ile)	1	rs769931559	n/a	n/a	n/a	VUS	LP
c.4296C>G	p.(Asn1432Lys)	1	rs761669740	n/a	n/a	n/a	LB	LP
c.53+56C>T (IVS1+56C>T)	p.(?)	1	rs140393487	n/a	n/a	n/a	n/a	LB
c.2490+44A>C (IVS14+44A>C)	p.(?)	1	rs375692108	n/a	n/a	n/a	n/a	LB
c.2909-93C>T (IVS17-93C>T)	p.(?)	3	rs144455881	n/a	n/a	n/a	n/a	LB
c.3469-100C>G (IVS21-100C>G)	p.(?)	2	rs946757675	n/a	n/a	n/a	n/a	LB
c.3964-86T>C (IVS24-86T>C)	p.(?)	1	rs1340773814	n/a	n/a	n/a	n/a	LB

Footnotes: *CFTR*, cystic fibrosis transmembrane conductance regulator; dbSNP, single-nucleotide polymorphism database; ^a^ based on current CFTR-France database (https://cftr.iurc.montp.inserm.fr/cftr/ (accessed on 15 June 2023)); ^b^ based on current CFTR2 database (https://cftr2.org/ (accessed on 15 June 2023)); functional effect of nucleotide change as predicted from ^c^ LOVD (https://databases.lovd.nl/shared/genes/CFTR (accessed on 15 June 2023)), ^d^ InterVar (https://wintervar.wglab.org/ (accessed on 15 June 2023)), and ^e^ VARSOME (https://varsome.com/ (accessed on 15 June 2023)) bioinformatics tools.

## Data Availability

Data are available from the corresponding authors and the first author upon request.

## Supplementary Materials

The following supporting information can be downloaded at: https://www.mdpi.com/article/10.3390/genes14081608/s1. Table S1: *CFTR* complex alleles identified in our cohort (transcript, NM_000492.4).
