# Supplementary material for: Next-Generation Sequencing for Screening Analysis of Cystic Fibrosis: Spectrum and Novel Variants in a South–Central Italian Cohort
Source: Genes (Basel). 2023 Aug 11;14(8):1608. doi: 10.3390/genes14081608 (PMC10454170; doi:10.3390/genes14081608)
Supplement: Supplementary file 1 [file genes-14-01608-s001.zip › genes-2545868-supplementary.pdf]

Supplementary Table S1. *CFTR* complex alleles identified in our cohort (transcript, NM_000492.4).

**N° of subjects**	**Protein change**	**Clinical significance**
8	p.(Gly576Ala)	Likely benign
	p.(Arg668Cys)	
1	p.(Gly576Ala)	Unknown
	p.(Arg668Cys)	
	p.(Arg75Gln)	
1	p.(Phe508del)	Unknown
	p.(Arg668Cys)	
1	p.(Phe508del)	Unknown
	p.(Asn1303Lys)	
1	p.(Ala455Val)	Unknown
	c.2620-15C>G	
1	p.(Ala455Val)	Unknown
	p.(Leu997Phe)	
1	p.(Arg31Cys)	Unknown
	p.(Ala455Val)	
1	p.(Arg75Gln)	Unknown
	p.(Ala455Val)	
1	c.2490+44A>C	Unknown
	p.(Ala455Val)	
1	p.(Leu967Ser)	Unknown
	p.(Glu1418Argfs*14)	
1	p.(Leu1077Pro)	Unknown
	p.(Asp192Gly)	

Footnotes: *CFTR*, cystic fibrosis transmembrane conductance regulator
